# Alpha-tubulin enhanced renal tubular cell proliferation and tissue repair but reduced cell death and cell-crystal adhesion

**DOI:** 10.1038/srep28808

**Published:** 2016-07-01

**Authors:** Juthatip Manissorn, Supaporn Khamchun, Arada Vinaiphat, Visith Thongboonkerd

**Affiliations:** 1 Medical Proteomics Unit, Office for Research and Development, Faculty of Medicine Siriraj Hospital, and Center for Research in Complex Systems Science, Mahidol University, Bangkok 10700, Thailand

## Abstract

Adhesion of calcium oxalate (CaOx) crystals on renal tubular epithelial cells is a critical event for kidney stone disease that triggers many cascades of cellular response. Our previous expression proteomics study identified several altered proteins in MDCK renal tubular cells induced by CaOx crystals. However, functional significance of those changes had not been investigated. The present study thus aimed to define functional roles of such proteome data. Global protein network analysis using STRING software revealed α-tubulin, which was decreased, as one of central nodes of protein-protein interactions. Overexpression of α-tubulin (pcDNA6.2-TUBA1A) was then performed and its efficacy was confirmed. pcDNA6.2-TUBA1A could maintain levels of α-tubulin and its direct interacting partner, vimentin, after crystal exposure. Also, pcDNA6.2-TUBA1A successfully reduced cell death to almost the basal level and increased cell proliferation after crystal exposure. Additionally, tissue repair capacity was improved in pcDNA6.2-TUBA1A cells. Moreover, cell-crystal adhesion was reduced by pcDNA6.2-TUBA1A. Finally, levels of potential crystal receptors (HSP90, HSP70, and α-enolase) on apical membrane were dramatically reduced to basal levels by pcDNA6.2-TUBA1A. These findings implicate that α-tubulin has protective roles in kidney stone disease by preventing cell death and cell-crystal adhesion, but on the other hand, enhancing cell proliferation and tissue repair function.

Until now, kidney stone disease is still a public health problem in almost all areas around the world. The disease causes substantial suffering and ultimately end-stage renal disease (ESRD). Unfortunately, the disease mechanisms remain poorly understood. Calcium oxalate (CaOx) is the major chemical component found in clinical stones^1^. This type of the stones can be originated from supersaturation of calcium and oxalate ions, leading to crystallization inside renal tubular fluid or urine^2^. CaOx crystals can then nucleate to form “stone nidus” and adhere directly onto apical surface of renal tubular epithelial cells^3^,^4^,^5^. Adhesion of crystals onto the cells is a critical event, which triggers many cascades of cellular response, e.g. cytotoxicity, injury, proliferation and apoptosis, that ultimately lead to kidney stone formation^6^,^7^. CaOx crystals also evoke inflammatory processes that can lead to fibrosis, loss of nephron and eventually ESRD^8^,^9^.

Even with the aforementioned knowledge, molecular mechanisms of the downstream cellular response remain largely unknown. From our previous expression proteomics study^7^, we have identified a number of proteins with altered levels in MDCK renal tubular cells in response to CaOx crystals. Those altered proteins were involved in various biological processes, i.e. ubiquitination pathway, signal transduction, cellular structure, purine biosynthesis, metabolic enzyme, retinol biosynthesis, cellular transportation, protein degradation, RNA metabolism, RNA binding protein, cell surface antigen, nucleic acid metabolism, antioxidant enzyme, chaperone, carrier protein, and protein biosynthesis. However, functional significance of those altered proteins had not been investigated. In the present study, we thus performed global protein network analysis of those altered proteins. Subsequently, overexpression of a protein, which was one of the central nodes of such protein-protein interactions network, was performed. Moreover, functional investigations were performed to address functional significance of the central-node protein and its associated partners in kidney stone disease.

## Results

### Global protein network analysis

**Figure 1:**
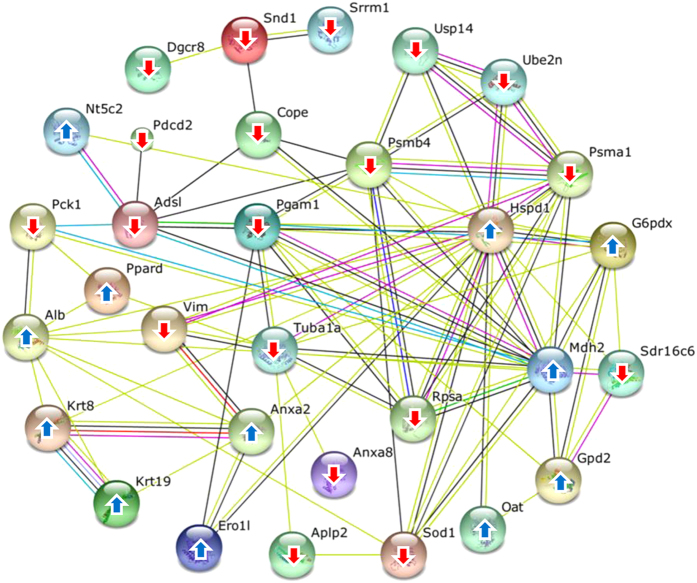
Global protein network analysis of altered proteins in MDCK renal tubular cells induced by CaOx crystals. All the altered proteins identified in our previous study^7^ were subjected to global protein network analysis using STRING tool (version 10) (http://string.embl.de/)^10^. Upward and downward arrows indicate up-regulation and down-regulation induced by the crystals, respectively. The connecting lines between protein nodes indicate protein-protein interactions.

From our previous expression proteomics study^7^, a number of differentially expressed proteins were identified in CaOx-treated MDCK cells. However, their functional roles in kidney stone disease had not been investigated. Our present study thus aimed to address functional significance of such altered proteins. First, they were submitted to global protein network analysis using STRING software (version 10) (http://string.embl.de/)^10^. The protein-protein interactions network demonstrated that α-tubulin was one of the central nodes of such protein-protein interactions (Fig. 1). We thus focused our attention on functional significance of α-tubulin in association with kidney stone formation.

### Α-tubulin overexpression (pcDNA6.2-TUBA1A) in MDCK cells and confirmation of α-tubulin level

**Figure 2:**
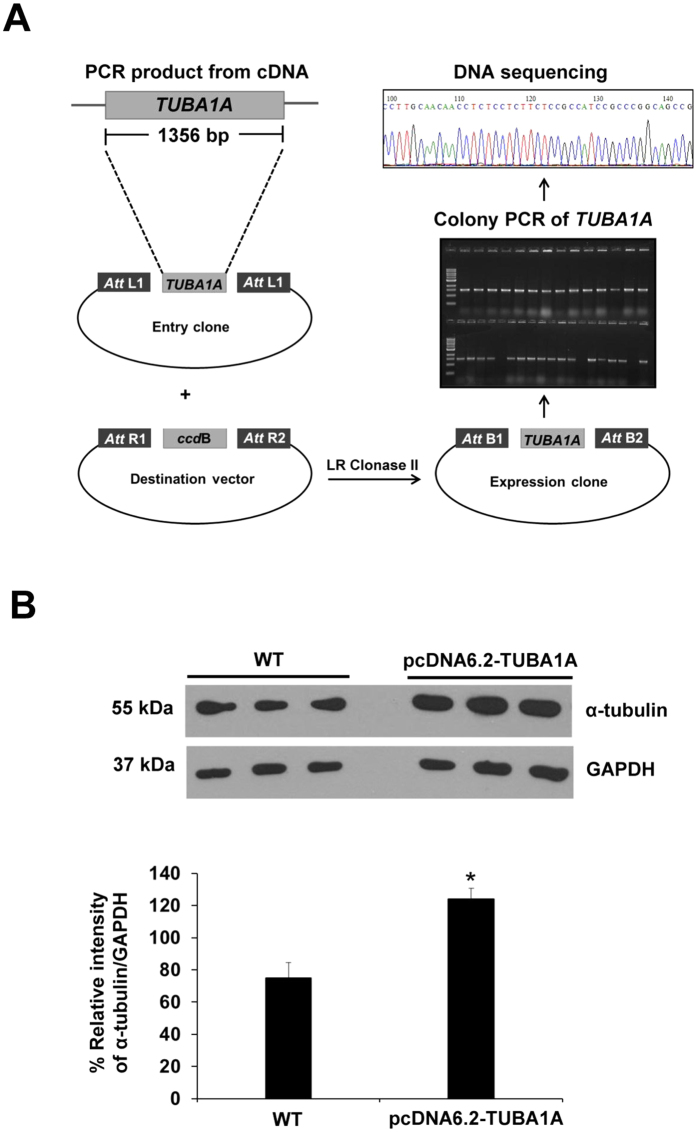
Overexpression of α-tubulin in MDCK cells. **(A)** Schematic diagram of α-tubulin overexpression (pcDNA6.2-TUBA1A) by Gateway Technology. **(B)** Efficacy of α-tubulin overexpression was confirmed by Western blot analysis. GAPDH served as the loading control. The data are reported as mean ± SEM (n = 3 independent experiments). *p < 0.05 vs. WT.

To address functional significance of α-tubulin, of which level was decreased in CaOx-treated MDCK cells, overexpression of α-tubulin was performed using Gateway Technology (Invitrogen). Figure 2A summarizes schematic approach of α-tubulin overexpression using this technology, which is based on pcDNA6.2-TUBA1A. Western blot analysis revealed that α-tubulin level was increased (approximately 1.5-fold) in pcDNA6.2-TUBA1A cells as compared to the unmodified (WT) cells, confirming that the overexpression of α-tubulin using this technique was successful (Fig. 2B).

### Effect of pcDNA6.2-TUBA1A on levels of α-tubulin, vimentin and ANXA2 in CaOx-treated cells

**Figure 3:**
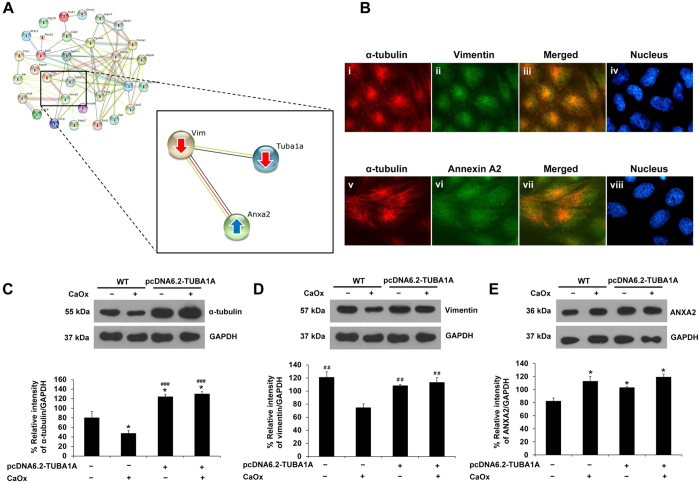
Effect of α-tubulin overexpression on levels of α-tubulin, vimentin and annexin A2 (ANXA2) in CaOx-treated cells. **(A)** Interactions or associations among α-tubulin, vimentin and ANXA2 are highlighted from the global protein interactions network. **(B)** Immunofluorescence study confirmed co-localizations (shown in yellow) of α-tubulin (in red) and vimentin (in green) (panels i-iv), as well as α-tubulin (in red) and ANXA2 (in green) (panels v-viii) (original magnification power was 1000X). **(C–E)** Whole cell lysate of MDCK cells with or without treatment with CaOx crystals for 48 h were subjected to Western blot analysis for α-tubulin **(C)**, vimentin **(D)**, and ANXA2 **(E)**. GAPDH served as the loading control. The data are reported as mean ± SEM (n = 3 independent experiments). *p < 0.05 vs. WT; ^##^*p* < 0.01 vs. WT + CaOx; ^###^*p* < 0.001 vs. WT + CaOx.

From STRING analysis, the protein-protein interactions network highlighted associations among cytoskeletal proteins (α-tubulin and vimentin) and a multi-function calcium-binding protein (annexin A2 or ANXA2) (Fig. 3A). Interestingly, α-tubulin directly interacted with vimentin, but indirectly interacted with ANXA2 via vimentin (Fig. 3A). Their associations were then validated by immunofluorescence co-staining. Figure 3B confirmed the co-localization between α-tubulin and its direct interacting partner, vimentin. Moreover, the data also revealed that α-tubulin co-localized with its indirect interacting partner, ANXA2 (Fig. 3B). Based on these direct and indirect interactions predicted by STRING analysis and confirmed by immunofluorescence study, levels of these interacting proteins were then examined upon exposure to CaOx crystals in both WT and pcDNA6.2-TUBA1A cells. Figure 3C shows Western blot data on α-tubulin that was decreased in WT cells after 48-h exposure to CaOx crystals, consistent to the expression proteomics data^7^. Overexpression of α-tubulin by pcDNA6.2-TUBA1A significantly increased α-tubulin level in the cells with and without CaOx treatment. Interestingly, while CaOx caused decrease in level of vimentin, pcDNA6.2-TUBA1A could restore the level of vimentin to its basal level as compared to both WT and pcDNA6.2-TUBA1A cells without CaOx treatment (Fig. 3D). For ANXA2, which was indirectly interacted with α-tubulin but directly interacted with vimentin, the data showed that the increase of ANXA2 upon CaOx treatment in WT was almost unaffected by pcDNA6.2-TUBA1A (Fig. 3E). This data was not unexpected as the overexpression of a target protein could affect its direct partner, whereas it was not necessary that those indirect partners would be affected by its overexpression (note that ANXA2 is a multi-function protein that also interacts with many other proteins in various biological pathways).

### Effect of pcDNA6.2-TUBA1A on cell death and proliferation

**Figure 4:**
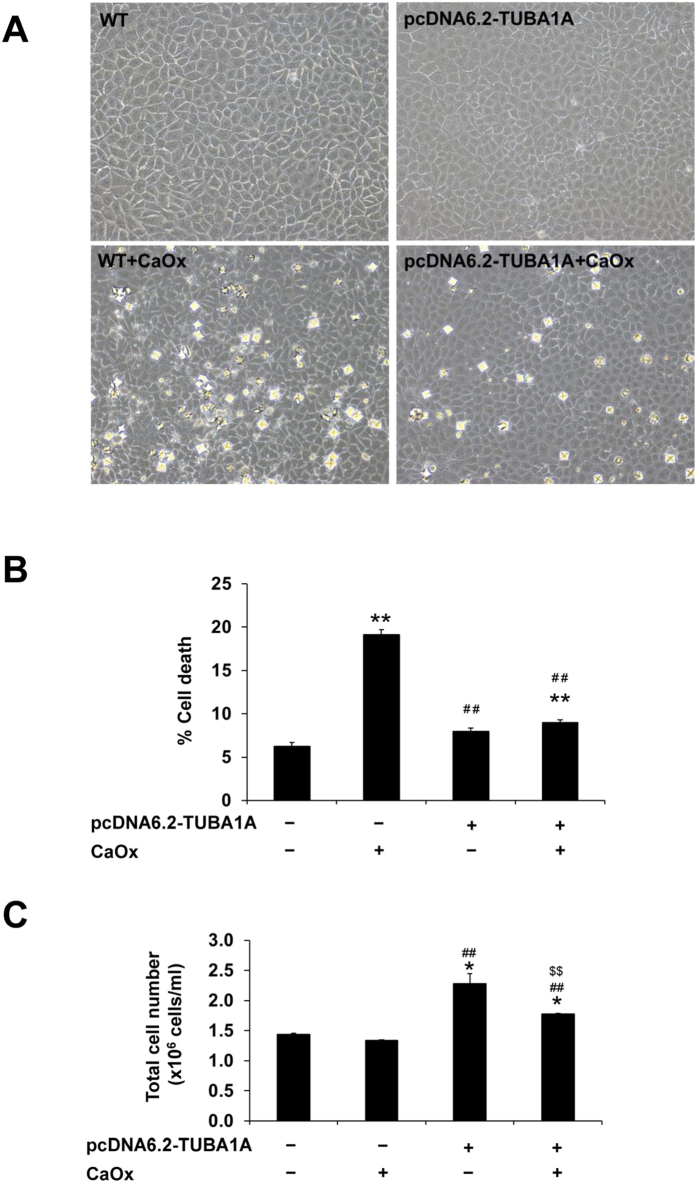
Effect of α-tubulin overexpression on cell death and proliferation. **(A)** Phase-contrast microscopy to evaluate cell morphology (original magnification power was 200X). **(B)** Cell death assay using Trypan blue staining. **(C)** Total cell number representing cell proliferation. The data are reported as mean ± SEM (n = 3 independent experiments). *p < 0.05 vs. WT; **p < 0.01 vs. WT; ^##^*p* < 0.01 vs. WT + CaOx; ^$$^*p* < 0.01 vs. pcDNA6.2-TUBA1A.

Cell morphology was observed under a phase-contrast microscope. Without CaOx treatment, morphology of WT and pcDNA6.2-TUBA1A cells looked normal without any significant differences observed (Fig. 4A). After 48-h treatment with CaOx crystals, the WT cells looked unhealthy with disruption of cell borders, whereas pcDNA6.2-TUBA1A cells remained normal (Fig. 4A). Cell death assay showed dramatic increase of cell death in WT cells exposed to CaOx crystals as compared to the untreated WT cells (Fig. 4B). pcDNA6.2-TUBA1A successfully reduced the percentage of cell death in the CaOx-treated cells to its basal level (Fig. 4B). In addition, we hypothesized that if α-tubulin overexpression could decrease cell death, it might also promote cell proliferation. To address this hypothesis, the total cell number was determined. The data showed that pcDNA6.2-TUBA1A could increase the total cell number in both untreated and CaOx-treated cells, as compared to the WT (note that the degree of such increase was slightly inferior in the CaOx-treated cells) (Fig. 4C).

### Effect of pcDNA6.2-TUBA1A on cell cycle

**Figure 5:**
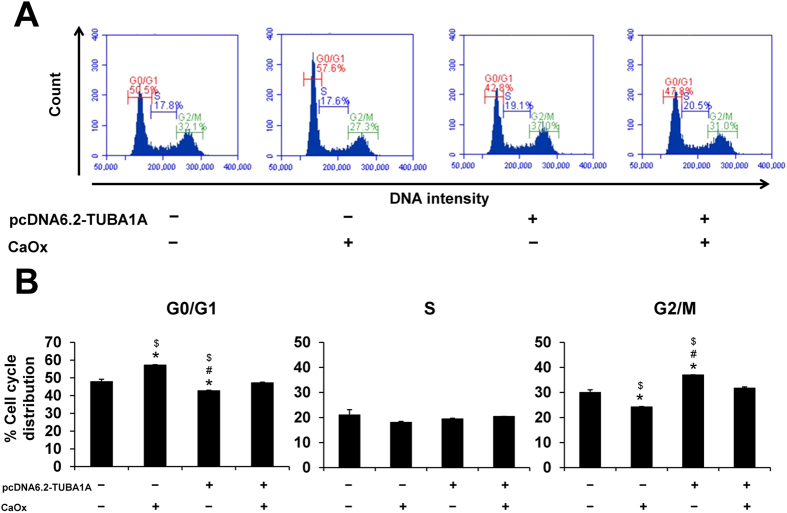
Effect of α-tubulin overexpression on cell cycle. Evaluation of cell cycle was performed by flow cytometry. (**A**) Representative flow cytometric data of cell cycles stained with propidium iodide. (**B**) Quantitative and statistical analyses of cell-cycle phases (G0/G1, S, and G2/M). The data are reported as mean ± SEM (n = 3 independent experiments). *p < 0.05 vs. WT; ^#^*p* < 0.05 vs. WT + CaOx; ^$^*p* < 0.05 vs. pcDNA6.2-TUBA1A + CaOx.

Cell cycle assay was performed using propidium iodide staining and analyzed by flow cytometry (Fig. 5A). Without CaOx treatment, pcDNA6.2-TUBA1A cells were shifted into G2/M or mitotic phase, while the CaOx-treated WT cells were found mainly in G0/G1 phase (Fig. 5B). Moreover, pcDNA6.2-TUBA1A could restore the cell cycle to normal after CaOx treatment (comparable to the untreated WT cells). These findings indicated that pcDNA6.2-TUBA1A enabled cells undergoing mitosis to promote cell division and had the protective role against cell cycle shift caused by CaOx crystals.

### Effect of pcDNA6.2-TUBA1A on tissue repair

**Figure 6:**
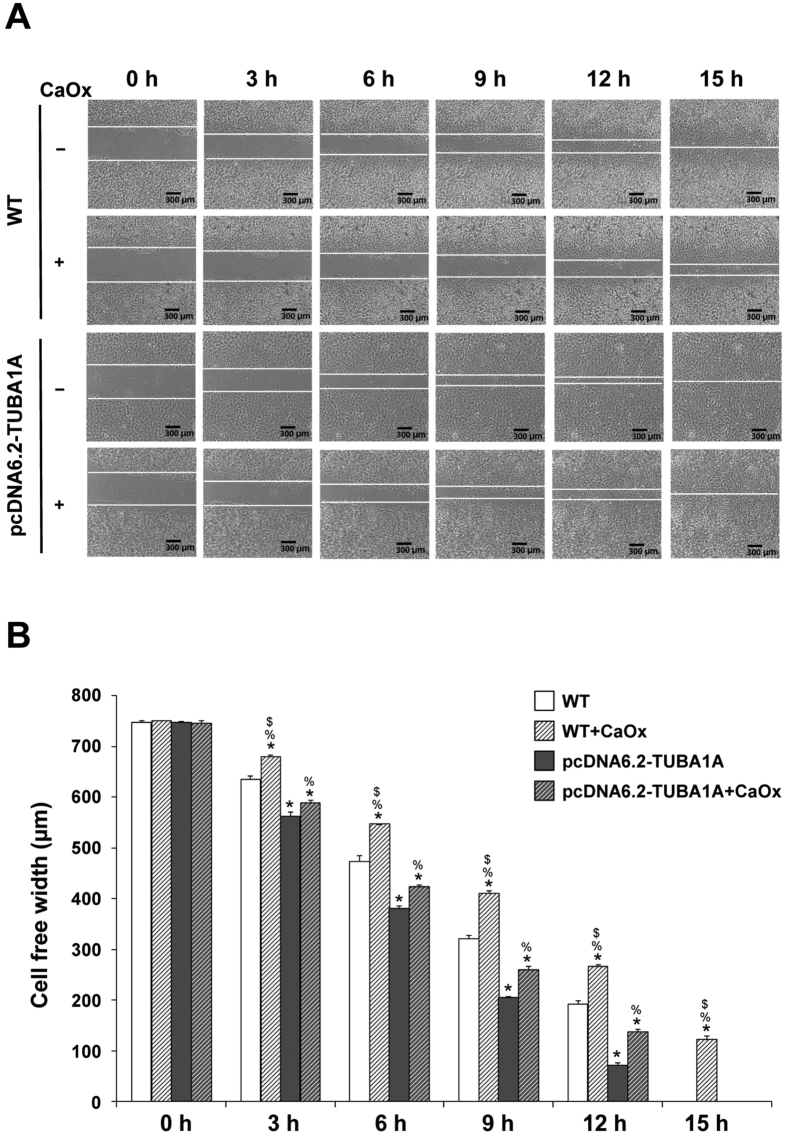
Effect of α-tubulin overexpression on tissue repair. Evaluation of tissue repair was done by using a scratch assay. (**A**) Representative images of cell-free width used for quantitative analysis (original magnification power was 40X). (**B**) Quantitative and statistical analyses of cell-free width at 0–15 h. The data are reported as mean ± SEM (n = 3 independent experiments). *p < 0.05 vs. WT; **p < 0.01 vs. WT; ^##^*p* < 0.01 vs. WT + CaOx; ^%^*p* < 0.05 vs. pcDNA6.2-TUBA1A; ^$^*p* < 0.05 vs. pcDNA6.2-TUBA1A + CaOx.

To evaluate tissue repair capacity, the cell monolayers were scratched by a 200-μl pipette tip to generate a cell-free gap. At basal time-point (0 h after scratch), the cell-free width was approximately 750 μm in all conditions (Fig. 6). Differences were detectable from 3–12 h after scratch – pcDNA6.2-TUBA1A could enhance tissue repair capacity of the cells at all time-points in corresponding groups. Moreover, both untreated and CaOx-treated pcDNA6.2-TUBA1A cells had better tissue repair capacity as compared to the untreated WT cells (Fig. 6). This data strengthened the importance of α-tubulin in tissue repair function.

### Effect of pcDNA6.2-TUBA1A on cell-crystal adhesion and expression of potential CaOx crystal receptors on apical membrane of MDCK cells

**Figure 7:**
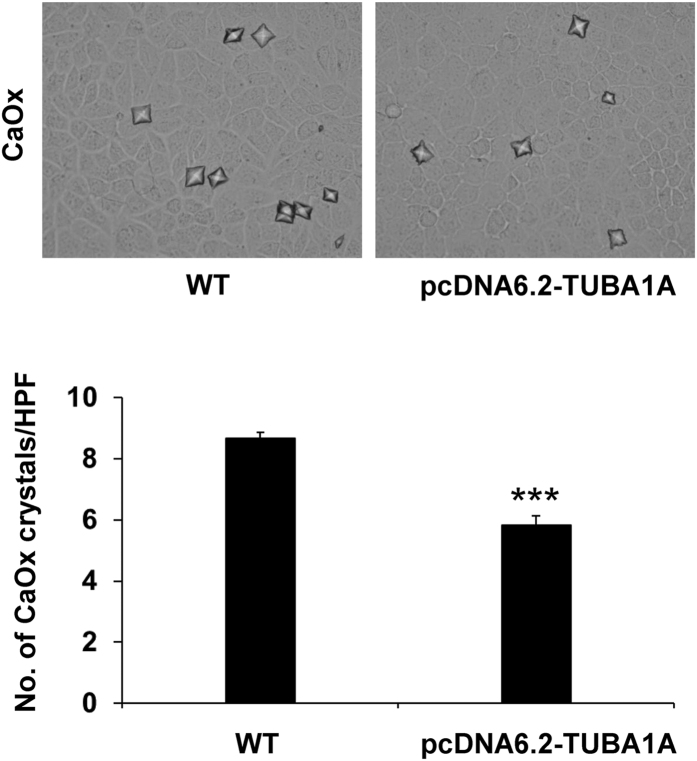
Effect of α-tubulin overexpression on cell-crystal adhesion. Upper panels show phase-contrast microscopic images of remaining crystals adhered on the surface of WT and pcDNA6.2-TUBA1A cells (original magnification power was 400X). Lower panel demonstrate the quantitative and statistical data. The data are reported as mean ± SEM (n = 6 independent experiments). ***p < 0.001 vs. WT.

Finally, the role of α-tubulin in a critical mechanism of kidney stone formation was evaluated. Crystal-cell adhesion assay revealed that pcDNA6.2-TUBA1A had significantly less degree of cell-crystal adhesion – there were significantly smaller numbers of the adherent crystals on the cell surface of pcDNA6.2-TUBA1A cells as compared to those of WT (Fig. 7). This data is the first dataset to demonstrate the protective role of α-tubulin in kidney stone formation.

**Figure 8:**
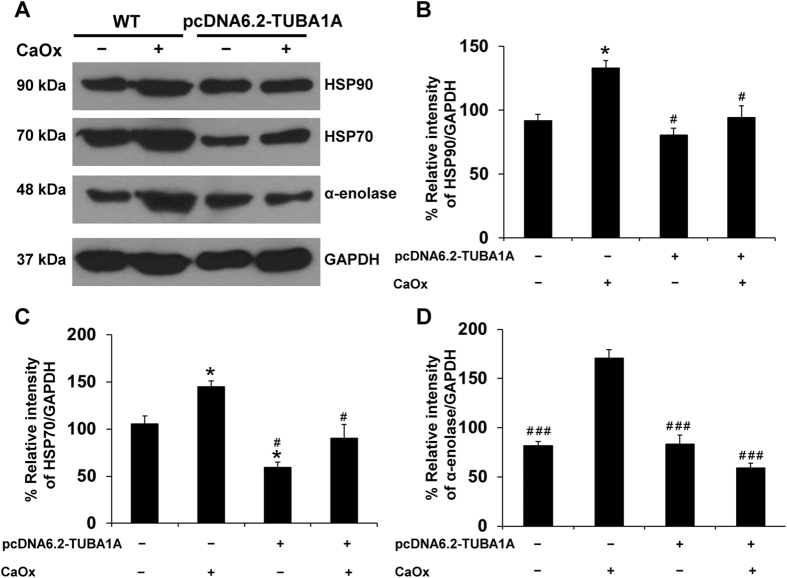
Effect of α-tubulin overexpression on expression of potential CaOx crystal receptors on apical membrane of MDCK cells. **(A)** Expression levels of potential CaOx crystal receptors, including HSP90, HSP70 and α-enolase, on apical membrane of polarized MDCK cells were investigated by Western blot analysis. GAPDH served as the loading control. **(B–D)** Quantitative data are reported as mean ± SEM (n = 3 independent experiments). *p < 0.05 vs. WT; ^#^*p* < 0.01 vs. WT + CaOx; ^###^*p* < 0.001 vs. WT + CaOx.

We then examined the possible mechanisms underlying such protective role of α-tubulin. Recently, we have reported a set of membrane proteins on apical membrane of MDCK polarized epithelial cells that might serve as CaOx crystal-binding molecules on the apical membrane or crystal receptors^11^. In the present study, we thus examined levels of some of those potential crystal receptors and found that levels of heat shock protein 90 (HSP90), HSP70, and α-enolase were significantly decreased on apical membrane of CaOx-treated pcDNA6.2-TUBA1A cells as compared to the CaOx-treated WT cells (Fig. 8). These data strengthen the protective role of α-tubulin in kidney stone formation via decreased expression of potential CaOx receptors on apical membrane of renal tubular epithelial cells.

## Discussion

From our previous study^7^, we have identified a number of proteins with significantly altered levels when the cells were exposed to CaOx crystals. In the present study, we further explored functional aspects of those altered proteins. From global protein network analysis using STRING tool (Fig. 1), protein-protein interaction network pointed out that α-tubulin played significant roles in response to CaOx crystal, which is the major causative chemical crystalline or solid composition inside kidney stone matrices. In expression proteomics work, α-tubulin was decreased by CaOx crystal exposure. To address functional significance of α-tubulin, overexpression that has been applied to several aspects of biology and disease models^12^,^13^, was thus performed and its efficacy was confirmed by Western blot analysis (Fig. 2).

Α-tubulin (55 kDa) is a cytoskeletal protein and major component of microtubules that plays a crucial role in regulation of cell shape, intracellular transport, cell motility, cell migration and cell division^14^,^15^. In addition, microtubule is important for tight junction preservation and restoration^16^. Depolymerization of microtubule can disrupt tight junction and reduce cellular adhesion and spreading^17^,^18^. Yap *et al*.^19^ have demonstrated that alterations in microtubule composition could affect the integrity of epithelial cell sheet. Moreover, emerging evidence has suggested that tubulin and microtubule-associated proteins may play roles in cellular stress response that favor survival of the cancer cells^20^.

Vimentin, another cytoskeletal protein, has been reported to play significant roles to maintain cell and tissue integrity^21^. It has been found to interact with several organelles, such as plasma membrane, lysosome, golgi, nucleus, and microtubules^22^. In our present study, we found that vimentin, of which level was also reduced by CaOx crystals^7^, directly interacted with α-tubulin. In addition, ANXA2 (a multi-function protein that has been previously documented as CaOx-binding molecules^9^,^23^) was also identified as a partner of the protein-protein interactions network. However, ANXA2 did not directly interact to α-tubulin while directly interacted with vimentin (Fig. 3A). We then validated their interaction by demonstrating their co-localizations (Fig. 3B). Their interactions were also confirmed by examination of their levels after the cells were exposed to CaOx crystals for 48 h. The data showed that while vimentin level had been dramatically influenced by α-tubulin overexpression (pcDNA6.2-TUBA1A) and could restore at its basal level when the cells were exposed with CaOx crystals, degree of the increased ANXA2 level induced by CaOx crystals was almost unaffected by pcDNA6.2-TUBA1A (Fig. 3D,E), consistent to the order or proximity of their interactions (Fig. 3A).

The scratch assay generally facilitates a study of cell migration, tissue reorganization, cell division^24^, and in our present study, tissue repair function. Α-tubulin overexpression was found to promote cell proliferation (Fig. 4), cell division (Fig. 5), and tissue repair (Fig. 6), mimicking the tumor-like phenotype. When cell-cell contact was disrupted by artificial wound, they tended to repair the wound through a combination of proliferation and migration^24^,^25^,^26^. This implied that α-tubulin overexpression promoted tissue repair by increased cell proliferation and cell migration. Moreover, our present study found that α-tubulin overexpression also increased expression of vimentin (Fig. 3). From previous evidence, vimentin has been reported to involve in tissue/wound repair in mice^27^. Therefore, it was plausible that the enhanced cell proliferation and tissue repair capacity of the pcDNA6.2-TUBA1A cells were from the combined effects of increased levels of both α-tubulin and vimentin.

CaOx crystals are injurious to the cell and may result to cytotoxicity. Many previous reports have shown that CaOx not only induced renal tubular cell injury but also enhanced crystal attachment on renal cell surface^5^,^28^,^29^. When crystals adhered on the epical cell surface, it could damage cell membrane^6^,^30^. To prove the hypothesis that α-tubulin overexpression could protect crystal attachment, cell-crystal adhesion assay was performed. The results confirmed that the degree of cell-crystal adhesion was significantly reduced by pcDNA6.2-TUBA1A (Fig. 7).

Furthermore, our previous studies reported that high-calcium and high-oxalate conditions could enhance crystal-binding proteins, such as HSP90, HSP70 and α-enolase, on apical membrane of MDCK cells^11^,^31^,^32^. To address whether these potential crystal receptors might be responsible for the reduction of cell-crystal adhesion, Western blotting was performed to measure levels of these proteins. The data nicely confirmed such hypothesis (Fig. 8).

In conclusion, our present study has demonstrated that overexpression of α-tubulin could protect renal tubular epithelial cells from cytotoxicity and cell death induced by CaOx crystals, while cell proliferation and tissue repair capacity were enhanced. Moreover, α-tubulin overexpression could reduce the degree of cell-crystal adhesion and decrease levels of potential crystal receptors (HSP90, HSP70, and α-enolase) on apical membrane of the cells that might be responsible for the reduction of cell-crystal adhesion. Taken together, α-tubulin has protective roles in kidney stone disease by preventing cell death and cell-crystal adhesion, but on the other hand, enhancing cell proliferation and tissue repair function.

## Materials and Methods

### Global protein network analysis

To obtain additional protein information for subsequent functional validation, all of the differentially expressed proteins identified from our previous study^7^ were subjected to global protein network analysis using STRING tool (version 10) (http://string.embl.de/)^10^. The predicted protein-protein associations were queried through experimentally derived physical protein interactions from literatures combining with the databases of curated biological pathway knowledge^10^.

### Cell cultivation

Madin-Darby canine kidney (MDCK) cells were maintained in minimum essential medium (MEM) supplemented with 10% FBS, 2 mM L-glutamine and 1.2% Penicillin G/Streptomycin (GIBCO, Invitrogen Corporation; Grand Island, NY) in a humidified incubator at 37 °C with 5% CO_2_. Polarization of the cells was achieved by using Transwells (0.4 μm pore size; Corstar; Cambridge, MA).

### RNA extraction and RT-PCR amplification of α-tubulin gene (*TUBA1A*)

To overexpress *TUBA1A* gene, the cDNA was prepared from MDCK cells. Briefly, MDCK cells were grown in 60-mm dishes and then harvested for total RNA extraction using Trizol reagent (Invitrogen, Life Technologies; Carlsbad, CA). The cDNA was then prepared using Super Script III (Invitrogen) and reverse transcription-PCR (RT-PCR) was performed using specific primers. PCR primers were designed for *TUBA1A* gene based on human sequence retrieved from CCDS database (accession no. CCDS8781) and the forward primer (5′-GCAACAACCTCTCCTCTTCG-3′) and the reverse primer (5′-TCCCTGTAAAAGCAGCACCT-3′) were used to amplify the entire *TUBA1A* gene. The PCR product was amplified using Phusion high fidelity DNA polymerase (New England BioLabs; Beverly, MA) and the amplification was carried out under the following conditions: a preliminary denaturation at 98 °C for 3 min, 34 cycles of denaturation at 98 °C for 30 s, annealing at 55 °C for 30 s, elongation at 72 °C for 1 min, and a final extension at 72 °C for 10 min. PCR product was separated by 1.2% agarose gel electrophoresis and detected by staining with ethidium bromide. The DNA bands were visualized using ChemiDoc MP Imaging System (Bio-Rad; Berkeley, CA).

### Cloning of the *TUBA1A* gene into expression vector and mammalian cell transfection

The 1.5-kb *TUBA1A* gene was cloned into an expression vector using Gateway Technology (Invitrogen). The entry clone was generated using pCR8/GW/TOPO vector (Invitrogen). The plasmid DNA was then extracted before subcloning into destination vector using Vivid colors pcDNA 6.2/EmGFP-Bsd/V5-DEST and LR clonase II enzyme (Invitrogen). Plasmid DNA was extracted and confirmed by DNA sequencing. The DNA sequence of *TUBA1A* was submitted to GenGank/EMBL/DDBJ (accession no. AB853091). Both controlled cells (WT) and α-tubulin-overexpressed cells (containing pcDNA6.2-TUBA1A vector) were transfected using Lipofectamin 2000 (Invitrogen) and 8 μg/ml blasticidin was added to generate the stable cell line. Overexpression of α-tubulin was confirmed by Western blot analysis.

### Western blot analysis

To validate overexpression of α-tubulin and to examine levels of its associated partners or related proteins, Western blot analysis was performed. Whole cell lysate or apical membrane protein fraction (isolation of apical membrane is detailed below) from MDCK cells were prepared in Laemmli’s buffer and resolved by 12% SDS-PAGE under reducing condition (with an equal amount of 30 μg/lane). The resolved proteins were then transferred onto a nitrocellulose membrane using a semi-dry transfer apparatus (GE Healthcare; Uppsala, Sweden) at 85 V. Non-specific bindings were blocked with 5% skim milk in PBS. The membrane was then incubated overnight at 4 °C with each of primary antibodies: mouse monoclonal anti-α-tubulin, mouse monoclonal anti-vimentin, goat-polyclonal anti-ANXA2, mouse monoclonal anti-HSP90, mouse monoclonal anti-HSP70, rabbit-polyclonal anti-α-enolase, or mouse monoclonal anti-GAPDH (all were purchased from Santa Cruz Biotechnology Inc.; Santa Cruz, CA and were diluted 1:1,000 in 1% skim milk/PBS). After washing with PBS three times, the membrane was further incubated with corresponding secondary antibody conjugated with horseradish peroxidase (1:2,000 in 1% skim milk/PBS; DAKO; Glostrup, Denmark) for 1 h at room temperature (RT) (set at 25 °C). The immunoreactive protein bands were visualized with SuperSignal West Pico chemiluminescence substrate (Pierce Biotechnology, Inc.; Rockford, IL) using autoradiogram.

### Immunofluorescence co-staining

To demonstrate protein co-localizations and to confirm protein-protein interactions predicted by STRING analysis, immunofluorescence study was performed. MDCK cells were cultivated on a cover slip for 24 h and the cells were fixed with 3.7% formaldehyde/PBS for 15 min and permeabilized with 0.1% triton X-100/PBS for 15 min. The cells were then incubated with mouse monoclonal anti-α-tubulin, rabbit polyclonal anti-vimentin, or goat-polyclonal anti-ANXA2 (all were purchased from Santa Cruz Biotechnology Inc. and were diluted 1:50 in 1% BSA/PBS) at 37 °C for 1 h. After washing, the cells were incubated with corresponding secondary antibody conjugated with Alexa 488 (in green; for α-tubulin) or Alexa 555 (in red; for vimentin and ANXA2) (Invitrogen-Molecular Probes; Burlinton, ON, Canada; all were diluted 1:500 in 1% BSA/PBS) at 37 °C for 1 h, whereas 0.1 μg/ml Hoechst dye (Invitrogen-Molecular Probes) was also co-incubated for nuclear staining. The cells were finally mounted with 50% glycerol/PBA and images were captured under an ECLIPSE 80i fluorescence microscope (Nikon; Tokyo, Japan).

### Preparation of CaOx crystals and exposure of MDCK cells to the crystals

CaOx crystals were prepared in an artificial urine as previously described^33^. Briefly, 125 ml of 25.08 mM CaCl_2_.2H_2_O was added into 250 ml of a buffer containing 19.26 mM tri-sodium citrate dihydrate (C_6_H_5_Na_3_O_7_.2H_2_O), 23.1 mM magnesium sulfate heptahydrate (MgSO_4_.7H_2_O) and 127.4 mM potassium chloride (KCl). The pH of the solution was adjusted to 6.5 using HCl. The solution was then incubated at RT for 15 min. Thereafter, 125 ml of 6.4 mM sodium oxalate (Na_2_C_2_O_4_) was added under a continuous stirring. The solution was incubated further at RT for 15 min. CaOx crystals were then harvested by centrifugation at 2,000 × *g* for 5 min. Supernatant was discarded and the crystals were resuspended in methanol. After another centrifugation at 2,000 × *g* for 5 min, methanol was discarded and the crystals were air-dried. After crystal generation and harvesting, the crystals were decontaminated with UV irradiation for 30 min. They were then added to a complete MEM medium (GIBCO, Invitrogen Corporation) to achieve a final concentration of 1,000 μg of crystals/ml of medium. MDCK cells were cultivated in complete MEM medium without or with crystals for 48 h in a humidified incubator at 37 °C with 5% CO_2_.

### Cell death and cell proliferation assays

Both WT and α-tubulin-overexpressed (pcDNA6.2-TUBA1A) cells with or without CaOx treatment were subjected to quantitative analysis of cell death and proliferation using trypan blue staining. Briefly, The cells were detached from the cultured well using 0.1% trypsin in 2.5 mM EDTA and immediately resuspended in MEM supplemented with 10% FBS to terminate trypsin activity. Aliquots of cell suspension were mixed with 0.4% trypan blue solution (GIBCO) and the cells were then counted using hemacytometer. Trypan blue-positive cells represented dead cells, whereas all the cells counted represented cell proliferation at 48 h after crystal exposure.

### Cell cycle assay

Both WT and α-tubulin-overexpressed (pcDNA6.2-TUBA1A) cells were inoculated in 60-mm culture dish. After 48-h incubation with or without CaOx crystals, cell cycle was analyzed and quantitated by flow cytometry after propidium iodide staining. Briefly, the cells were collected by trypsinization, fixed and permeabilized with ice-cold 70% ethanol, and incubated on ice for 30 min. Thereafter, the cells were washed twice and resuspended in PBS containing 100 μg/ml RNase A (Sigma-Aldrich; St. Louis, MO). After incubation for 30 min, the cells were stained with propidium iodide (BD Biosciences; San Jose, CA) at RT in the dark for 10 min. The stained cells were finally analyzed for their DNA content using BD Accuri C6 flow cytometer (BD Accuri, Beckman Coulter; Fullerton, CA). At least 10,000 events per each sample were evaluated and the cell cycle histograms were generated by BD Accuri™ C6 software in order to quantify the percentage of cells in G0/G1, S and G2/M phases.

### Tissue repair assay

Tissue repair capacity of the cells was evaluated by using a scratch method. Briefly, both WT and α-tubulin-overexpressed (pcDNA6.2-TUBA1A) cells were inoculated in 6-well plate. After 48-h treatment with or without CaOx crystals incubation, the cell monolayers were scratched along the culture well diameter using a 200-μl pipette tip to create a cell-free area. After washing with PBS to remove debris and detached cells, the cultures were further maintained in a humidified incubator at 37 °C with 5% CO_2_. At indicated time-points (0, 3, 6, 9, 12 and 15 h after the scratch), scratched wound size was monitored using BioStation CT (Nikon Corp.; Tokyo, Japan). The captured images were submitted to Tarosoft Image framework v.0.9.6 (Nikon) to accurately measure the cell-free width.

### Cell-crystal adhesion assay

Both WT and α-tubulin-overexpressed (pcDNA6.2-TUBA1A) cells were inoculated in 6-well plate and maintained for 48-h. Thereafter, the culture medium was removed and the cells were washed with PBS twice. Crystal-cell adhesion was initiated by the addition of 10% FBS-supplemented MEM containing crystals into each well. The cells were further incubated in a humidified incubator at 37 °C with 5% CO_2_ for 30 min. Thereafter, the cells were vigorously washed using PBS five times to remove the unbound crystals. Finally, the remaining crystals adhered on the cell surface were counted in 20 randomized high power fields (HPF) per culture well.

### Isolation of apical membrane of MDCK cells by peeling method

Both WT and α-tubulin-overexpressed (pcDNA6.2-TUBA1A) cells at a density of approximately 5.0–7.5 × 10^4^ cells/ml were seeded and grown on prewetted collagen-coated permeable polycarbonate membrane in Transwells for four days. The culture medium was refreshed every other day. After 48-h treatment with or without CaOx crystals, the cell monolayers were washed with PBS three times to remove the unbound crystals. Apical membrane of the polarized MDCK cells was isolated by a peeling method recently established^34^. Briefly, Whatman filter paper (0.18-mm-thick, Whatman International Ltd.; Maidstone, UK) pre-wetted with deionized water was placed onto the polarized cell monolayer. After a 5-min incubation period, the filter paper was peeled out and the apical membranes retained at the filter paper surface were harvested by rehydration in deionized water and gentle scrapping. The apical membrane-enriched fraction was then lyophilized. Dried apical membrane was solubilized in 1X Laemmli’s buffer and quantitated by Bradford’s method using Bio-Rad Protein Assay (Bio-Rad Laboratories; Hercules, CA). The recovered proteins were then subjected to Western blot analysis for potential crystal receptors.

### Statistical analysis

Quantitative analyses were done in at least triplicates of independent experiments unless stated otherwise. All quantitative data are presented as mean ± SEM. Comparisons between the two groups of samples were performed using unpaired Student’s *t*-test, whereas multiple comparisons of more than two groups of samples were performed using one-way analysis of variance (ANOVA) with Tukey’s post-hoc test. *P* values less than 0.05 were considered statistically significant.

## Additional Information

**How to cite this article**: Manissorn, J. *et al*. Alpha-tubulin enhanced renal tubular cell proliferation and tissue repair but reduced cell death and cell-crystal adhesion. *Sci. Rep.* **6**, 28808; doi: 10.1038/srep28808 (2016).
